# Stepwise Activation of the ATR Signaling Pathway upon Increasing Replication Stress Impacts Fragile Site Integrity

**DOI:** 10.1371/journal.pgen.1003643

**Published:** 2013-07-18

**Authors:** Stéphane Koundrioukoff, Sandra Carignon, Hervé Técher, Anne Letessier, Olivier Brison, Michelle Debatisse

**Affiliations:** Institut Curie UMR 3244, Université Pierre et Marie Curie (Paris 06), CNRS Paris, France; Baylor College of Medicine, United States of America

## Abstract

Breaks at common fragile sites (CFS) are a recognized source of genome instability in pre-neoplastic lesions, but how such checkpoint-proficient cells escape surveillance and continue cycling is unknown. Here we show, in lymphocytes and fibroblasts, that moderate replication stresses like those inducing breaks at CFSs trigger chromatin loading of sensors and mediators of the ATR pathway but fail to activate Chk1 or p53. Consistently, we found that cells depleted of ATR, but not of Chk1, accumulate single-stranded DNA upon Mre11-dependent resection of collapsed forks. Partial activation of the pathway under moderate stress thus takes steps against fork disassembly but tolerates S-phase progression and mitotic onset. We show that fork protection by ATR is crucial to CFS integrity, specifically in the cell type where a given site displays paucity in backup replication origins. Tolerance to mitotic entry with under-replicated CFSs therefore results in chromosome breaks, providing a pool of cells committed to further instability.

## Author Summary

Accurate genome duplication is crucial at each cell generation to maintain genetic information. However, replication forks routinely face lesions on the DNA template and/or travel through sequences intrinsically difficult to replicate, such as common fragile sites (CFS). To help the fork to proceed, the cells have evolved the DNA damage checkpoint that senses different types of damage and triggers well-adapted cellular responses. We have studied the DNA damage response of human lymphoblastoid cells and normal fibroblasts to various levels of fork slowing. We showed that a two- to ten-fold reduction of fork speed leads to global chromatin recruitment of sensors and mediators of the ATR pathway without substantial activation of Chk1, ATM or p53. Analysis of the phenotype of cells depleted of ATR or Chk1 and submitted to moderate levels of stress shows that ATR, but not Chk1, is crucial to CFS integrity. We propose a model explaining how fork speed thresholds direct fine-tuned checkpoint responses that protect genome integrity without blocking cell cycle progression upon moderate replication fork impediment. Tolerance to mitotic entry with under-replicated CFSs therefore results in chromosome breaks, providing a pool of cells committed to further instability.

## Introduction

Accurate genome duplication is required at each cell generation to maintain genetic information. However, mammalian genomes contain regions that challenge the replication process, such as common fragile sites (CFS). CFSs are loci that recurrently exhibit breaks on mitotic chromosomes following moderate slowing of replication fork movement [1]. To date, there is a consensus considering that such stresses delay completion of CFS replication more than the rest of the genome, and that breaks occur at under-replicated sequences upon chromosome condensation at mitotic onset. This delay was believed to result from replication fork blockage arising when forks encounter secondary structures formed at particular nucleotide sequences, notably AT-rich repeats [1]. However, the instability of *FRA3B*, the most active CFS in human lymphocytes, was recently shown to result from paucity in initiation events along a large region overlapping the most instable part of the site. This paucity forces replication forks emanating from flanking origins to cover long distances before merging in late S or G2 phases, leaving the sites incompletely replicated upon fork slowing [2]. Strikingly, *FRA3B* is weakly fragile in fibroblasts, in which initiation events are evenly distributed all along the locus [2]. Conversely, the two major CFSs in fibroblasts, that are not fragile in lymphocytes, display origin paucity in fibroblasts and a normal distribution of initiation events in lymphocytes [3]. Thus, the tissue-dependent organization of replication initiation controls the epigenetic setting of CFSs [4].

CFSs are a recognized source of the genomic instability driving oncogenesis from early steps of the process [5]. Indeed, CFS instability was repeatedly observed in pre-neoplasic lesions [5], [6], [7]. How pre-neoplasic cells, that generally retain wild-type checkpoints, escape surveillance by the DNA damage response (DDR) remains unclear. Central to DDR are two related protein kinases, ATM and ATR, that respectively sense double strand breaks (DSB) and RPA-coated single stranded DNA (ssDNA) accumulated upon fork slowing [8]. ATR and ATM activation then leads to phosphorylation of a large panel of substrates, including Chk1 and Chk2, which triggers a second wave of phosphorylations that amplifies and spreads the signal [9]. Among these downstream targets is the major tumour suppressor p53, a transcription factor that integrates signals from many different pathways [10]. Not surprisingly, inactivation of key DDR components leads to various diseases, including cancer [11].

In vertebrate cells, like in yeasts, the ATR/Mec1 pathway was mostly studied under conditions imposing a complete block to fork progression. Among other effects, such stresses lead, in *cis*, to stabilization of damaged replication forks and, in *trans*, to delayed mitotic onset [12]. In contrast, little is known about the cell response to moderate stresses such as treatments with low concentrations of aphidicolin, a well-known inhibitor of DNA polymerases, commonly used to induce breaks at CFSs. Several reports suggested that the frequency of breaks at CFSs increases in cells deprived of ATR, TopBP1, Hus1 or Chk1 [4]. However, while the role of ATR has been largely confirmed, notably *in vivo* in human patients and in mutant mice [13], [14], the impact of other proteins, including Chk1, in the maintenance of CFS integrity remains more controversial.

Here we compared the response of human lymphoblastoid cells and normal fibroblasts to various levels of fork slowing. We showed that a two- to ten-fold reduction of fork speed (called below moderate stress conditions) leads to global chromatin recruitment of sensors and mediators of the ATR pathway without substantial activation of Chk1, ATM or p53. Analysis of the phenotype of cells depleted of ATR or Chk1 and submitted to moderate levels of stress shows that ATR, but not Chk1, is crucial to fork protection and CFS integrity specifically in cell types where the site is fragile. These observations shed light on how pre-neoplastic cells continue cycling under inappropriate conditions.

## Results

### Chromatin loading of sensors and mediators of the ATR pathway upon fork slowing

**Figure 1:**
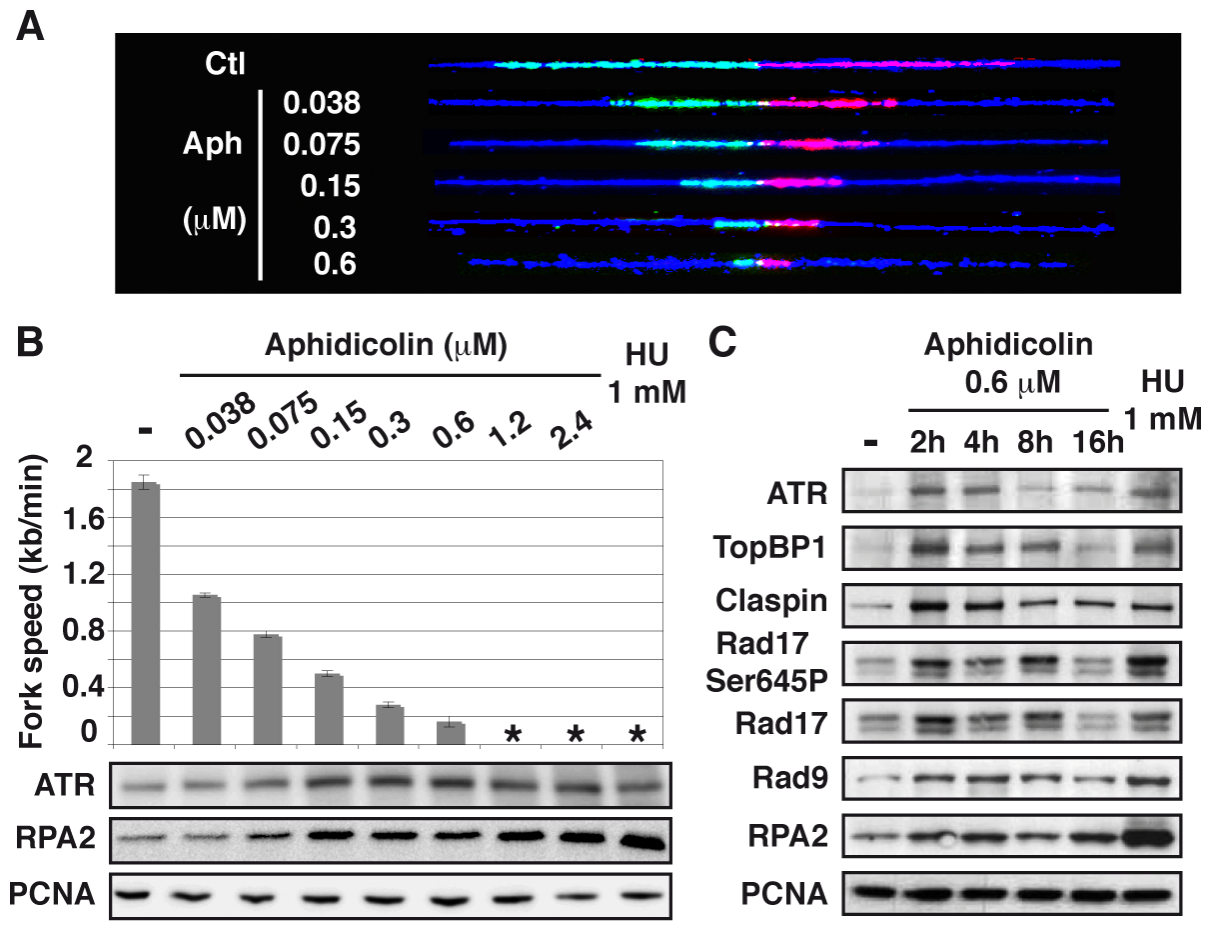
Moderate fork slowing triggers chromatin loading of sensors and mediators of the ATR pathway. (A) Measurement of replication fork speed. Typical examples of combed DNA molecules displaying replications forks in control cells (Ctl) and cells treated with aphidicolin. Cells were pulse-labelled with IdU then CldU before DNA was purified and combed (see Methods). The analogues were revealed on stretched molecules as previously described [44] and fork speed was determined by measuring the length of IdU (red) and CldU (green) tracks. Blue: counterstaining of the DNA used to select unbroken IdU and CldU tracks (B) Mean fork speed (upper panel), kb per min ± standard error of the mean (s.e.m.), and chromatin recruitment of checkpoint proteins (lower panel). Mean fork speed and chromatin recruitment of ATR and RPA2 were studied after 4 h of treatment with the indicated aphidicolin concentrations or 1 h of treatment with HU 1 mM. Asterisks indicate that fork speed cannot be measured. (C) Western blot analysis of chromatin extracts from exponentially growing cells, untreated (-) or treated as indicated.

We used DNA combing to determine how increasing concentrations of aphidicolin impact fork movement in JEFF cells (B lymphocytes immortalized by Eptsein-Barr virus) and MRC-5 cells (normal embryonic human fibroblasts) (Figure 1A). In untreated JEFF cells, forks progress at approximately 1.85 kb/min. In cells grown with aphidicolin 1.2 or 2.4 µM or with HU 1 mM, fork movement is too slow to be accurately measured with the labelling conditions we used. These treatments are considered below to block fork progression. For aphidicolin concentrations between 0.038 and 0.6 µM, the medians of fork speeds range between 1 and 0.2 kb/min (Figures 1B upper panel and S1), which is considered as moderate speed reduction. Similar results were obtained upon treatment of MRC-5 cells (Figure S2A).

The status of the ATR pathway was determined by western blot analysis of chromatin-bound ATR and RPA2 in cells treated for 4 h with different concentrations of aphidicolin or HU 1 mM (Figure 1B lower panels). We found that chromatin loading of ATR starts to increase in cells treated with aphidicolin 0.075 µM and reaches a maximum at 0.15 µM, namely when fork speed is reduced approximately by a factor of two. Noticeably, the amount of chromatin-bound RPA2 also starts to increase upon treatment with aphidicolin 0.075 µM, but remains lower in cells treated with up to 2.4 µM of the drug than in cells grown with HU 1 mM. The study of MRC-5 cells also shows that a two-fold decrease in fork speed triggers recruitment of ATR to the chromatin and that, like in JEFF cells, ATR binding behaves essentially as an all-or-nothing phenomenon (Figure S2B).

A time course analysis of the status of the ATR pathway in JEFF cells treated with aphidicolin 0.6 µM shows that ATR, TopBP1, Claspin, RPA2 and Rad9, a subunit of the 9-1-1 complex, are rapidly loaded on the chromatin (Figure 1C). Noticeably, Rad17 is loaded and phosphorylated on Ser645, a recognized ATR phosphorylation site [15]. We observed that the amount of chromatin-bound proteins decreases upon prolonged treatment, a phenomenon previously observed by others [16], [17]. Short treatments with aphidicolin 0.6 µM or HU 1 mM lead to comparable levels of recruitment for all proteins but RPA2, which chromatin amount remains stable and lower in the presence of aphidicolin than in the presence of HU. Thus, chromatin loading of checkpoint sensors and mediators of the ATR pathway surprisingly appears poorly correlated to the amount of chromatin-bound RPA.

**Figure 2:**
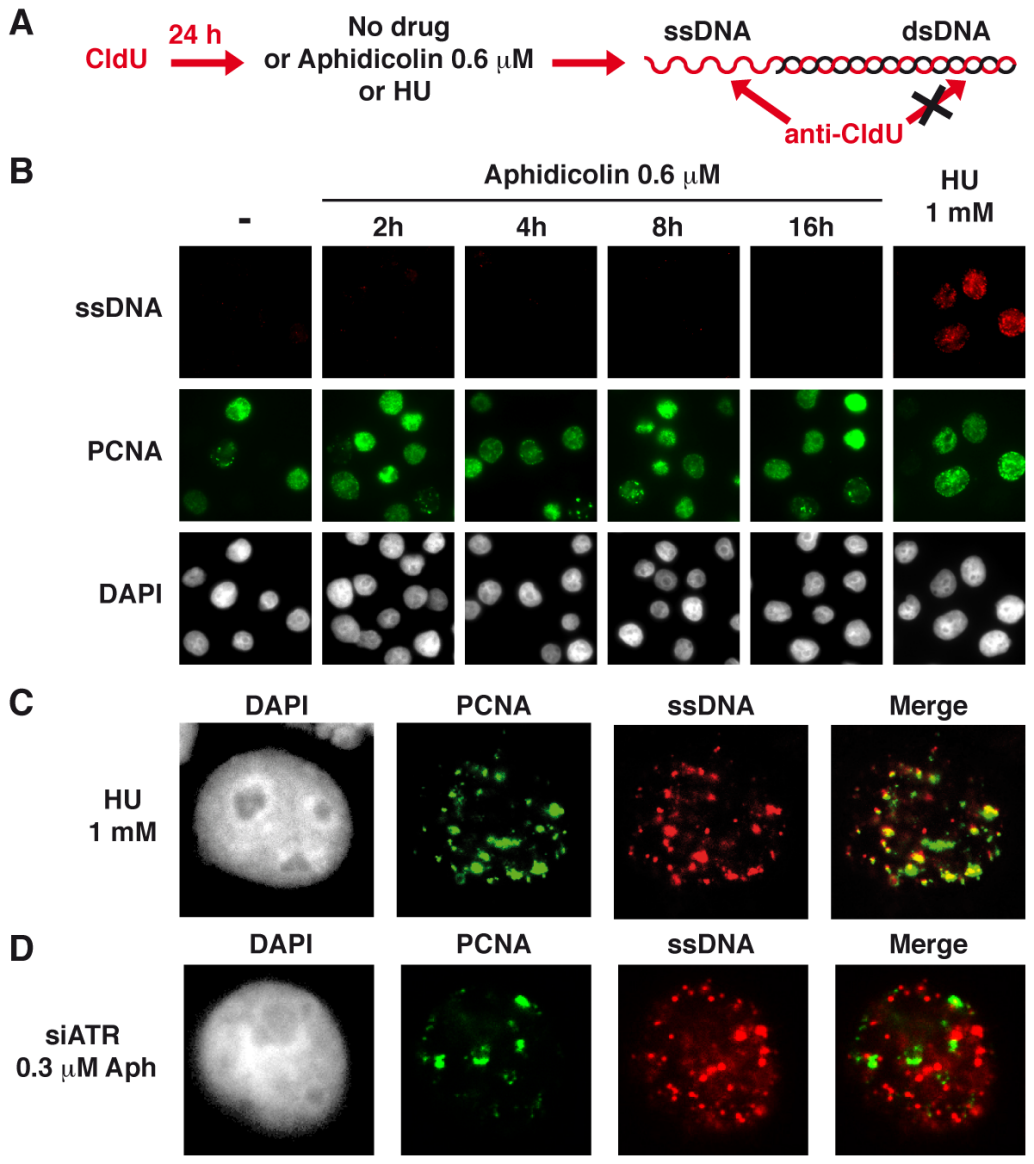
Moderate fork slowing is not associated with formation of ssDNA foci. (A) ssDNA detection scheme. Cells hemi-substituted throughout their genome after growth in the presence of CldU for about 1.5 cell generations were treated or not with aphidicolin or HU for different periods of time prior to fixation. CldU was immuno-detected without DNA denaturation, which only permits visualization of substituted and single-stranded regions. (B) Co-detection of ssDNA (red) and chromatin bound PCNA (green) by immunostaining of untreated cells (-) and cells treated as indicated. Nuclei were counterstained with DAPI. (C) Typical pattern of PCNA and CldU foci in cells treated with HU 1 mM during 1 h. (D) Typical pattern of PCNA and CldU foci in cells depleted of ATR and treated with 0.3 µM of aphidicolin for 4 h.

To reinforce this conclusion, we studied ssDNA accumulation in JEFF cells treated as above using the procedure schematized in Figure 2A. As expected, no CldU labelling was observed in untreated JEFF cells (Figure 2B). Following 1 h of treatment with HU 1 mM, 104 out of 110 (95%) cells in S-phase, identified by the presence of PCNA foci, display CldU foci while cells not in S-phase remain unlabelled. In addition, these CldU foci generally co-localize with PCNA foci (Figure 2C), showing that ssDNA forms at blocked forks. Strikingly, CldU foci were absent from the vast majority of S-phase cells following up to 16 h of treatment with aphidicolin 0.6 µM. Thus, under moderate speed reduction, the amount of sensors and mediators of the ATR pathway loaded on the chromatin is not proportional to the amount of RPA-coated ssDNA exhibited at the forks.

### Several ATR targets, including Chk1, are not activated upon moderate fork slowing

**Figure 3:**
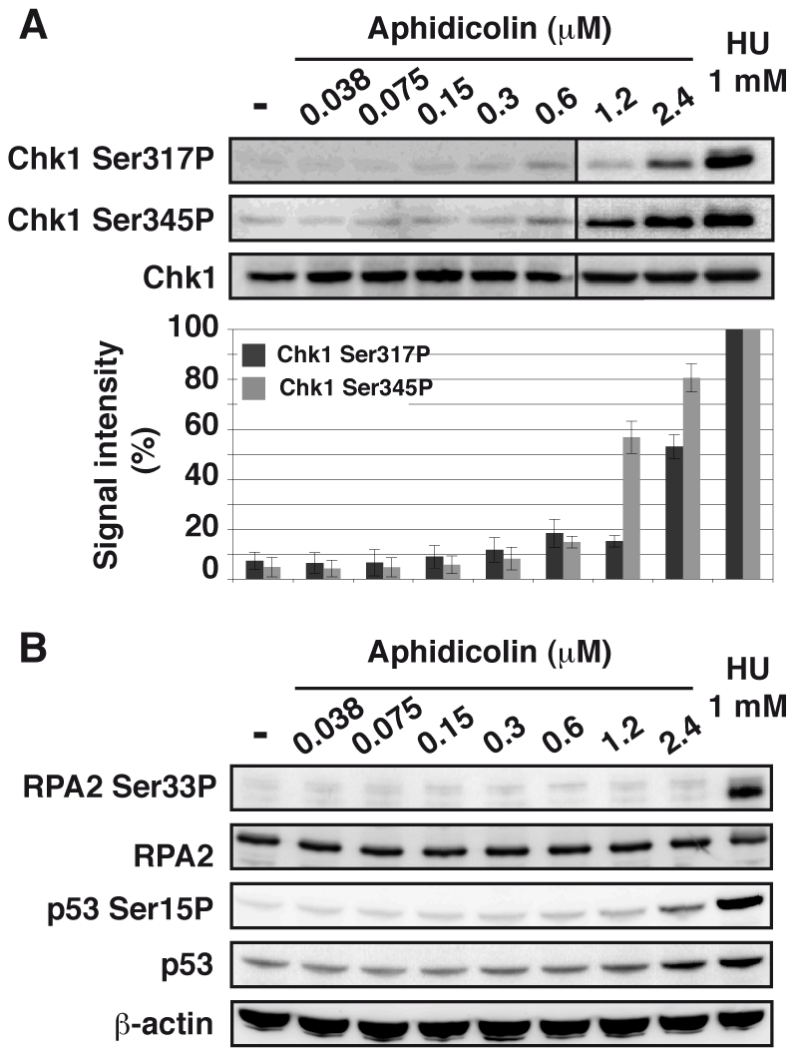
Moderate fork slowing does not trigger phosphorylation of ATR targets. (A) Western blot detection of Chk1-Ser317 and Ser345 phosphorylations in total extracts of cells treated 4 h with aphidicolin or 1 h with HU 1 mM (upper panel). Quantification of Chk1-Ser317P and -Ser345P from 3 independent experiments (lower panel). Results (mean ± s.e.m) are expressed as percentage of the level of phosphorylation found in cells treated with 1 mM HU. Lanes 1–6 and lanes 7–9 correspond to different blots. (B) Western blot detection of RPA2-Ser33 phosphorylation, p53 and p53-Ser15 phosphorylation in total extracts of cells treated for 4 h with aphidicolin or 1 h with HU 1 mM. Loading control: β-actin.

Chk1 phosphorylation on Ser317 and Ser345 was analyzed (Figure 3A). Both residues appear phosphorylated in JEFF cells treated with aphidicolin 2.4 µM or HU 1 mM but not, or weakly, in cells treated with up to 0.6 µM of aphidicolin. Chk1 status was confirmed by western blot analysis of extracts from S-phase cells (Figure S3A) and by immunofluorescence (Figure S3B). Similar observations were made with MRC-5 cells (Figure S2C). These results agree with some reports [17], [18] but others found Chk1 phosphorylated in some cancer cell lines upon treatment with aphidicolin 0.6 µM [19]. These discrepancies most probably reflect differences in genetic backgrounds leading to cell-type variations in aphidicolin sensitivity. Unfortunately, the absence of fork speed measurement in previous works prevents further comparison of the results.

In addition, we observed that phosphorylation of p53 on Ser15 and of RPA2 on Ser33 remains undetectable under moderate replication stress (Figure 3B). Therefore, chromatin-bound ATR fails to trigger activation of several DDR effectors under these conditions.

### Moderate fork speed reduction does not activate the ATM pathway

**Figure 4:**
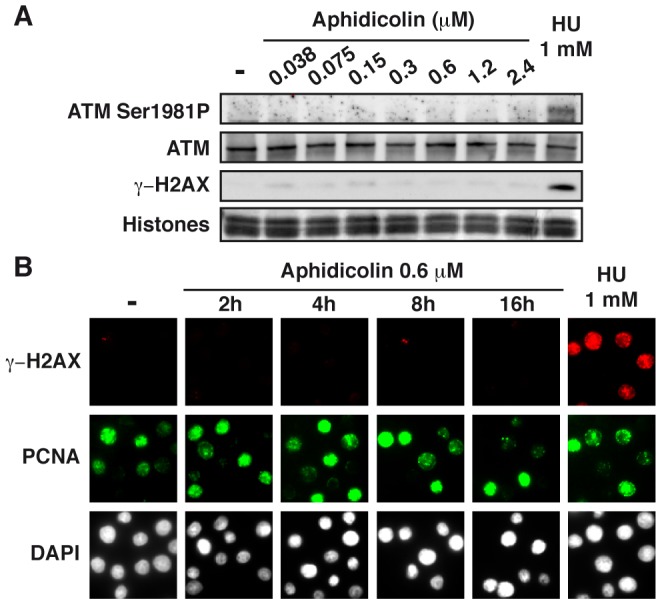
Moderate fork slowing triggers neither γ-H2AX foci nor ATM activation. (A) Western blot analysis of ATM-Ser1981 phosphorylation and γ-H2AX in exponentially growing cells, untreated (-) or treated as indicated. (B) Immunostaining of γ-H2AX together with chromatin-bound PCNA in untreated cells (-) and in cells treated with aphidicolin 0.6 µM for the indicated periods of time, or for 1 h with HU 1 mM.

To further analyze the cellular response to replication stress, we studied the status of the ATM pathway (Figure 4A). We found that a slight phosphorylation of ATM on Ser1981 starts to appear upon treatment with aphidicolin 2.4 µM, but remains much lower than in cells treated with HU 1 mM. Chromatin accumulation of ATM and phosphorylation of histone variant H2AX (γH2AX) occur only in cells treated with HU 1 mM. Time course analyses of ATM (Figure S3C) and H2AX (Figures 4B and S3D) in cells treated with aphidicolin 0.6 µM confirmed these observations. Thus, the ATM pathway is not triggered in cells experiencing moderate stress for up to 16 h.

### Moderate fork speed reduction results in Chk1-independent decrease in mitotic flow

**Figure 5:**
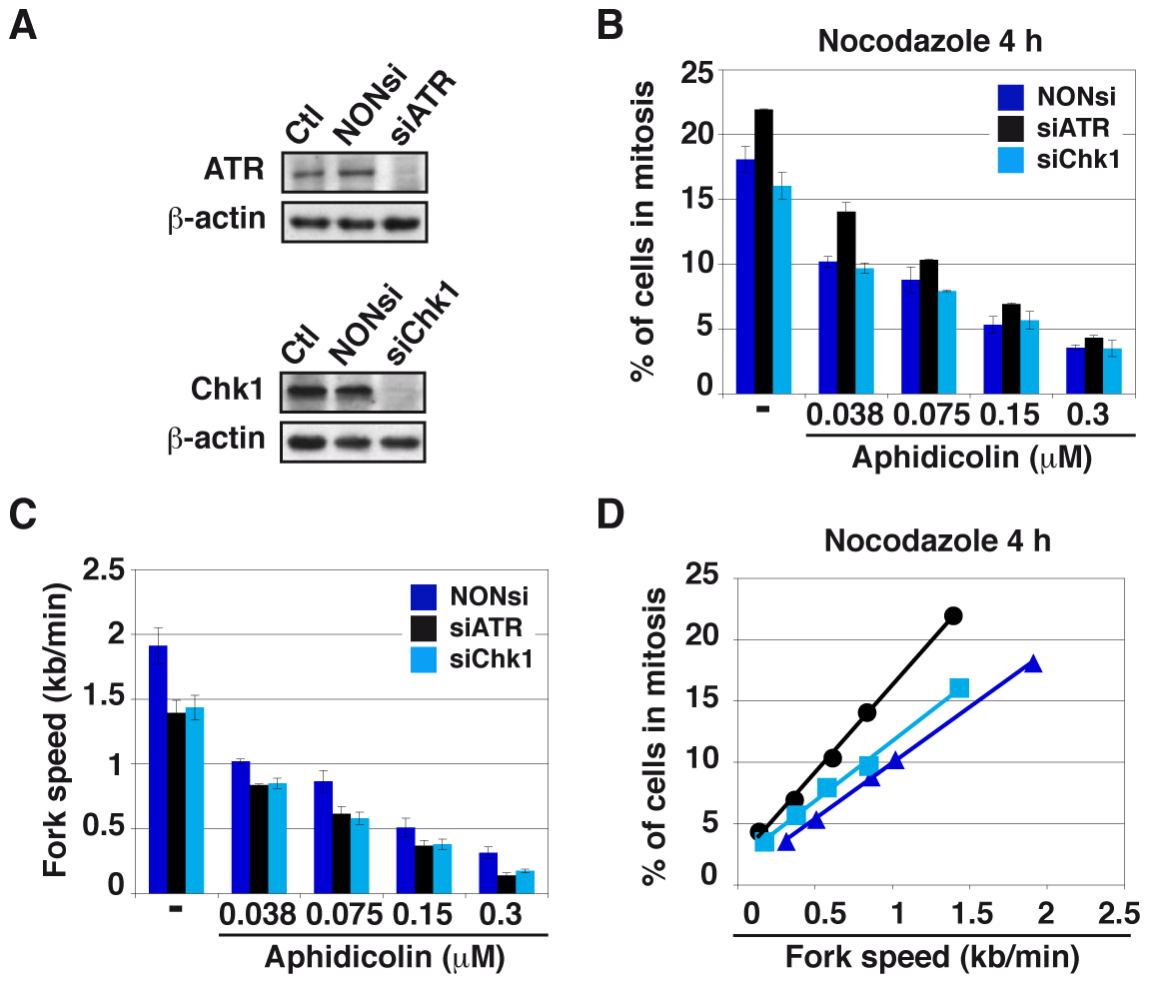
Moderate fork slowing decreases mitotic flow independently of ATR and Chk1. (A) Western blot analyses of total cell extracts 48 h post-transfection with a NONsi RNA or siRNAs specific to ATR (left panel) or Chk1 (right panel). Loading control: β-actin. (B) The percentages of cells in mitosis were measured by flow cytometry with MPM2 immuno-detection 48 h post-transfection with the indicated RNAs. Cells were treated for 4 hr with aphidicolin, then for 4 hr with both aphidicolin and nocodazole to block mitotic exit. Aphidicolin concentrations are indicated. (C) Measurement of replication speed in each condition of transfection and treatment. (D) Correlations between fork speed and the percentage of cells in mitosis. Data presented in B and C were used to plot the percentage of cells in mitosis against fork speed.

The fact that moderate stresses fail to activate the whole DDR cascade raises the question as whether mitotic onset is restrained under these conditions. The mitotic flow was determined following treatment with various aphidicolin concentrations and nocodazole to block mitotic exit, in DDR-proficient cell populations and in populations of cells depleted of either ATR or Chk1 by RNA silencing, (Figure 5A). We found that the percentages of JEFF cells entering mitosis decrease upon treatment with increasing aphidicolin concentrations in the three genetic backgrounds and that only ATR depletion modestly impacts these percentages (Figure 5B). Similar results were obtained in the presence of z-VAD fmk, a pan-caspase inhibitor that blocks apoptosis (Figure S3E). Thus, the deficit in mitotic cells we observed upon aphidicolin treatment does not result from mitotic death but rather from delayed mitotic entry.

We then determined how mitotic flow correlates with the degree of fork slowing. In good agreement with previous works [20], [21], we observed that ATR or Chk1 depletion “*per se*” reduces fork speed by a mechanism not yet elucidated. Not surprisingly, aphidicolin treatment further reduces fork movement (Figure 5C). Plotting the percentage of mitotic cells against fork speed in the different conditions reveals a linear relationship between the two parameters, regardless of the transfection conditions (Figures 5D and S3F). In addition, the curve obtained for Chk1-depleted cells aligns with that of control cells, indicating that the decrease in mitotic flow resulting from Chk1 depletion is completely accounted for by fork slowing. Therefore, Chk1 plays no direct role in the control of mitotic onset under these conditions, which agrees with the absence of Chk1 phosphorylation upon moderate fork speed reduction. The curve corresponding to ATR-depleted cells does not strictly align with the other two, which suggests that ATR plays a role, though modest, in the control of mitotic entry. The prominent mechanism that correlates mitotic flow to fork speed in these conditions is presently unknown.

### ATR, but not Chk1, is crucial for chromosome stability upon moderate fork slowing

**Figure 6:**
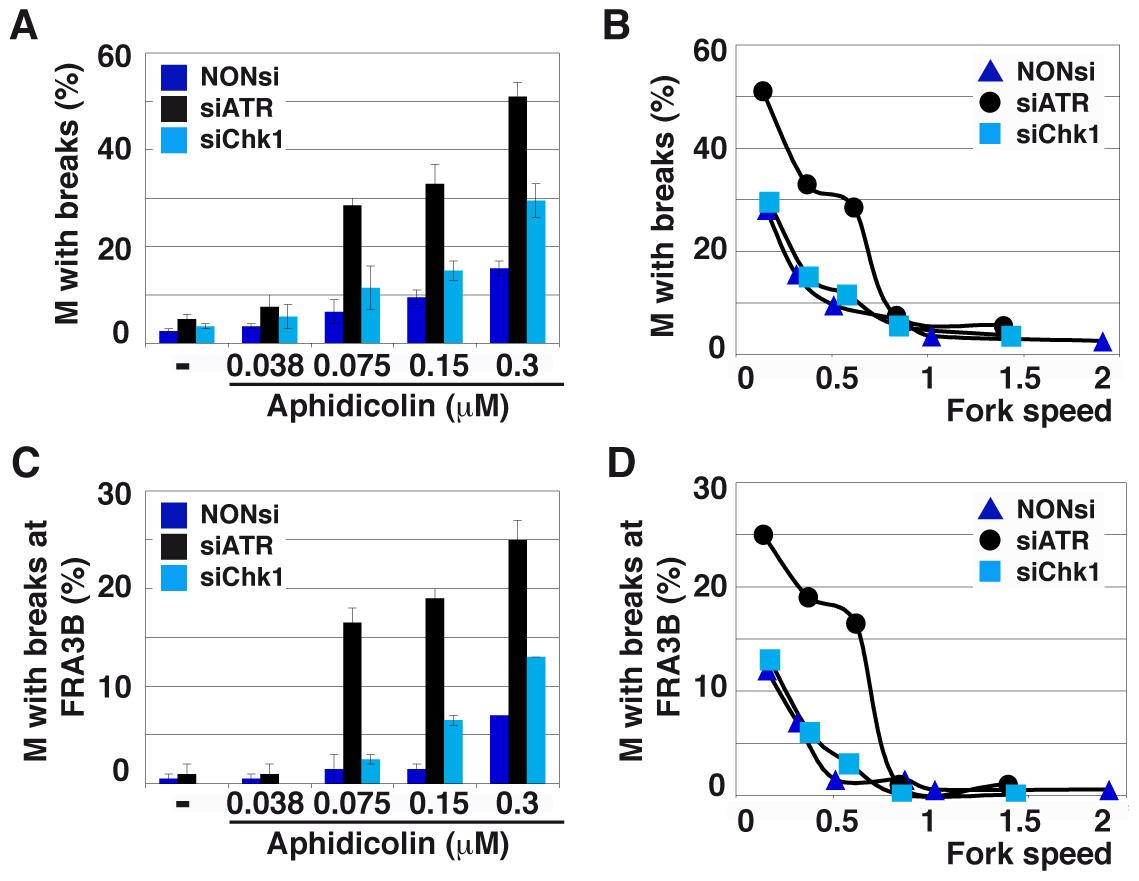
ATR depletion compromises genome stability. (A) Quantification of metaphases (M) with breaks in cells transfected as above and treated for 16 h with the indicated aphidicolin concentrations. Mean ± s.e.m are presented. (B) Correlations between replication fork speed and chromosome instability. The data presented in A and Figure 5C were used to plot the percentage of metaphase plates with chromosome breaks against fork speed in each condition of transfection and treatment. (C) Breaks occurring at *FRA3B* quantified as in C. (D) Correlations between replication speed and *FRA3B* instability evaluated as in B.

To evaluate the contribution of the ATR pathway to chromosome maintenance under moderate stress, we determined the percentage of metaphase plates displaying chromosome breaks in DDR-proficient cells and in cells depleted of either ATR or Chk1 following 16 h of treatment with various aphidicolin concentrations (Figures 6A and S4A). We found that this percentage is the highest in cells depleted of ATR, although Chk1 depletion also alters chromosome stability. The percentage of metaphases displaying chromosome breaks was then plotted against fork speed for the different conditions of cell depletion and treatment (Figure 6B). The curve obtained for Chk1-depleted cells is identical to that of control cells, indicating that the increase in chromosome breakage resulting from Chk1 depletion is completely accounted for by fork slowing. Strikingly, the curve for ATR-depleted cells coincides with the other two only when fork speed stands higher than 0.85 kb/min. Further slowing leads to percentages of cells with broken chromosomes greatly exceeding those observed in control or Chk1-depleted cells.

Breaks at CFSs usually account for most of the breaks induced by low aphidicolin concentrations. We thus used fluorescent in situ hybridization (FISH) (Figure S4B) to determine the percentage of metaphase plates displaying chromosome breaks at *FRA3B* (Figures 6C and 6D), the most active CFS in JEFF cells [2]. In either transfection conditions, breaks at *FRA3B* represent approximately 50% of total breaks and evolve like total breaks. Thus, ATR plays a major role in the maintenance of CFS stability when fork speed falls below a threshold of 0.85 kb/min. This value is consistent with the threshold for chromatin recruitment of ATR (Figure 1B). Noticeably, this high level of CFS instability is not explained by the weak effect of ATR depletion on mitotic entry (Figure 5D).

### CFSs co-localize with ssDNA foci in ATR-depleted and moderately stressed cells

**Figure 7:**
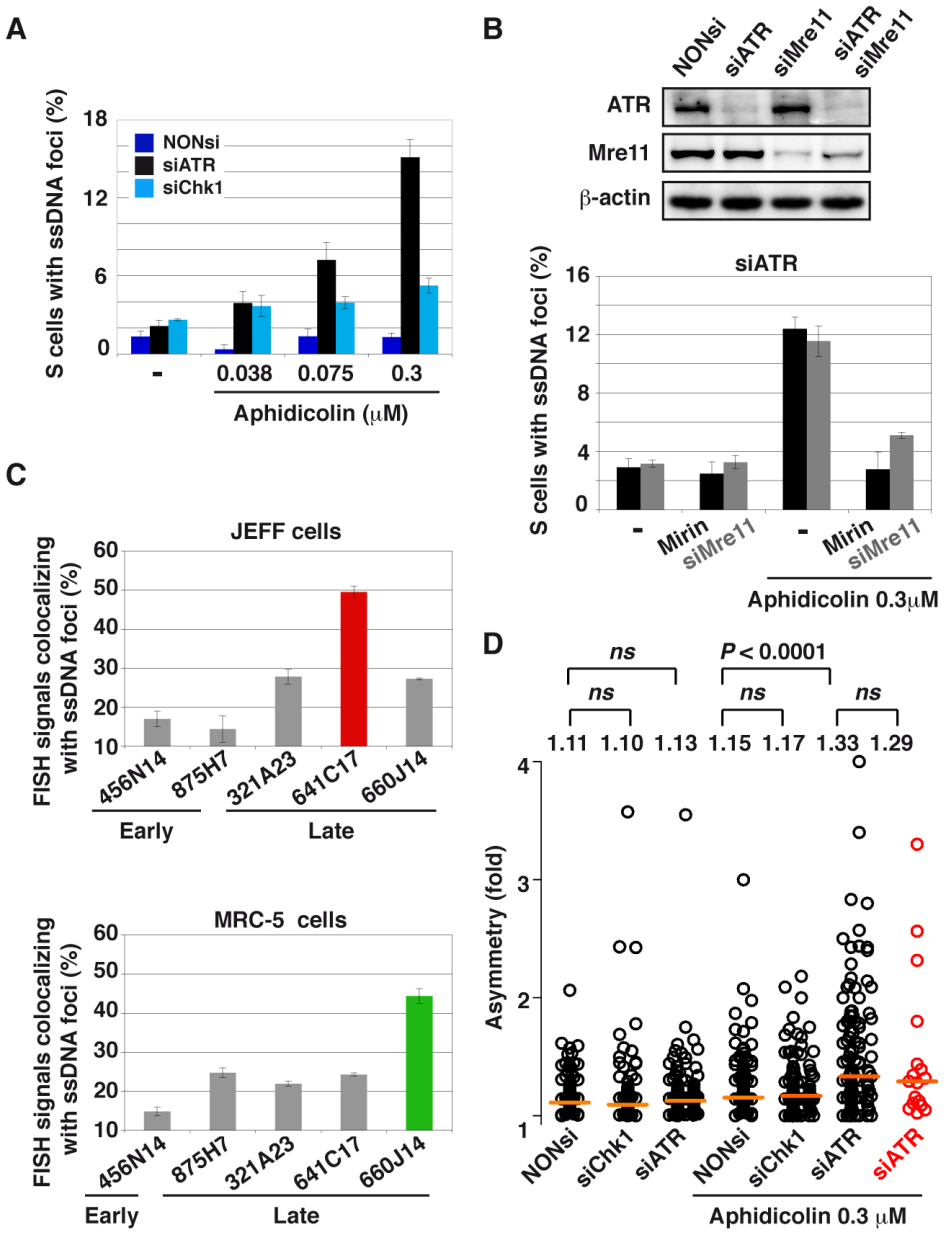
ATR-depletion enhances formation of ssDNA foci and fork asymmetry. (A) Percentage of S-phase cells with ssDNA foci in the indicated conditions of transfection and aphidicolin treatment. Mean ± s.e.m are presented. (B) Upper panel: Western blot analyses of total cell extracts 48 h post-transfection with a NONsi RNA or siRNAs specific to ATR or/and Mre11. Loading control: β-actin. Lower panel: percentage S-phase cells (mean ± s.e.m) displaying ssDNA foci in populations of cells depleted of ATR and treated or not with aphidicolin 0.3 µM. Mre11 status is indicated (siMre11 or treatment with Mirin: 100 µM). (C) Percentage of FISH signal co-localizing with ssDNA foci in cells depleted of ATR and treated with 0.3 µM aphidicolin. The immunostaining of ssDNA and PCNA was combined with FISH with BAC probes. Upper panel: JEFF lymphoblastoid cells, the BACs probe early replicating regions (875H7 and 456N14) and late replicating regions (321A23, 660J14, and 641C17 that corresponds to *FRA3B*, the major CFS in lymphocytes). Lower panel: MRC-5 fibroblasts, BAC 456N14 probe for an early replicating region, other BACs correspond to late replicating regions (875H7, 321A23, 641C17, and 660J14 which corresponds to the major CFS in fibroblast mapping at 3q13.3). (D) Distributions of fork asymmetry in cells transfected and treated as indicated. Forks travelling in the *FRA3B* locus are represented by red circles (*n* = 17) and in the bulk genome by black circles (untreated: NONsi, *n* = 128; siCHk1, *n* = 103; siATR, *n* = 147; with aphidicolin: NONsi, *n* = 121; siCHk1, *n* = 132; siATR, *n* = 123). Horizontal orange lines represent the medians of fork distributions. Medians and *P* values are indicated above the distributions (not significant, *ns*).

To determine whether break frequencies in the different genetic contexts correlate with the presence of ssDNA at the fork, we studied cells displaying foci of ssDNA and/or PCNA in the three conditions of transfection (Figure 7A). In the absence of aphidicolin treatment, we found that the percentage of cells displaying foci is low (approximately 2%) in either condition. Upon treatment with aphidicolin 0.3 µM, some 2% of DDR-proficient and 5% of Chk1-depleted cells exhibit CldU foci. This percentage reaches 15% in ATR-depleted cells and those foci strikingly do not co-localize with PCNA foci (Figure 2D), suggesting that ssDNA takes place at collapsed forks. To determine whether these foci result from resection of collapsed forks by the Mre11-Rad50-Nbs1 (MRN) complex, the cells were depleted of Mre11 by RNA silencing or treated with mirin, an inhibitor of Mre11 nuclease activity [22]. Strikingly, compared to ATR depleted cells treated with mirin 100 µM or depleted of Mre11, ATR-depleted cells treated with both aphidicolin 0.3 µM and mirin or co-depleted of ATR and Mre11 show similar percentages of cells with ssDNA foci (Figure 7B). These results suggest that ssDNA foci we observed result from Mre11-mediated resection of collapsed forks. This conclusion agrees with the results of a recent work showing that MRN activity leads to the formation of RPA foci in checkpoint deficient U2OS cells [23].

We then asked whether ssDNA foci formed in ATR-depleted cells treated with aphidicolin 0.3 µM co-localize with CFSs (Figure S4C). We found that *FRA3B* co-localizes more often with ssDNA foci (50%) than non-fragile regions replicating late (25%) or replicating early (15%) (Figure 7C). The relative co-localization of CFSs and ssDNA foci was also studied in MRC-5 fibroblasts where *FRA3B* is not fragile. In these cells the major CFS lies at 3q13.3 [3]. We found that this CFS also associates at higher frequency with ssDNA foci (45%) than other tested sequences, including *FRA3B* that behaves like non-fragile and late replicating sequences (Figure 7C). Noticeably, the non-fragile region identified by BACs 875H7, that replicates early in JEFF cells and late in MRC-5 cells [24], displays a percentage of co-localization with ssDNA foci that reflects its replication timing in each cell type, 12% and 24% respectively. The frequency of co-localization of a sequence with ssDNA foci is thus timing-dependent and, in the case of CFSs, correlates with their level of fragility in the tissue under study.

### Forks collapse and/or stall similarly along FRA3B and in the bulk genome

Finally, we asked whether the preferential association of *FRA3B* with ssDNA foci in JEFF cells reflects the fact that forks collapse or stall more often along the site than in the bulk genome. Collapse and/or stalling should lead to asymmetrical forks, namely to individual forks presenting unequal IdU and CldU tracks [2]. Therefore, we calculated asymmetry as the ratio of the longest to the shortest track in cells treated or not with aphidicolin 0.3 µM and depleted or not of ATR or Chk1 (Figures 7D and S5). At the genome-wide level, fork asymmetry increases significantly only in cells depleted of ATR and treated with aphidicolin. We also studied fork asymmetry along *FRA3B* in the latter conditions. Strikingly, the distributions and the medians of fork asymmetries along *FRA3B* and in the bulk genome appear remarkably similar.

## Discussion

We determine here how various levels of fork speed reduction impact DDR activation and genome integrity in human lymphoblastoid and fibroblastic cells. Surprisingly, ATR is loaded on the chromatin at roughly similar levels whether forks are completely blocked or only slowed by a factor of 2 to 3. This loading thus behaves as an all-or-nothing phenomenon. Other sensors and mediators of the ATR pathway accumulate on the chromatin with similar kinetics while a modest increase in RPA-coated ssDNA is seen in cells experiencing moderate stress. These results suggest a model in which speed thresholds dictate the DDR status. Upon moderate fork impediment, stretches of RPA-coated ssDNA may be generated at a small fraction of the forks, which would escape detection by the techniques we used. Fork impediment may occur at random in the genome, or preferentially along regions difficult to replicate such as micro- and mini-satellites [8], [25], [26], telomeric repeats [27], [28], [29], [30], [31] or highly transcribed genes [32], [33], [34], [35]. We postulate that blocked forks elicit an alarm signal that commits cells to take steps against replisome disassembly, notably by recruiting sensors and mediators of the ATR pathway at all on-going forks.

ATR contributes importantly to the stabilization of blocked forks in the yeast *S. cerevisiae* [36], [37], [38] and in higher eukaryotes [36]. We show here that ATR is also crucial to stabilize forks experiencing moderate slowing. Indeed, ssDNA foci form at high frequency in S-phase cells depleted of ATR and treated with low doses of aphidicolin. Those ssDNA foci do not co-localize with PCNA foci, which strikingly contrasts with the co-localization of ssDNA and PCNA foci we observed in DDR-proficient cells upon fork blockage. In addition, we observed that Mre11 depletion or Mre11 inhibition by mirin suppresses ssDNA foci formation in cells depleted of ATR and treated with aphidicolin. Together, these observations show that moderate stresses strongly impact fork stability in ATR-deficient cells, then resection of collapsed forks by the Mre11 nuclease activity gives rise to large amounts of ssDNA that form the foci we observe.

Although phosphorylation of Chk1, p53 or RPA2 does not occur in DDR-proficient cells submitted to moderate stress, the observation that Rad17 is phosphorylated indicates that chromatin-bound ATR is active. In addition, neither formation of γ-H2AX foci nor activation of ATM and/or Chk2 takes place under these conditions. How DDR is committed to complete activation remains unknown. Considering the many physiological situations that may lead to global or local replication fork slowing, such adaptation of the checkpoint response to the degree of stress might be crucial to cell proliferation, but this tolerance is detrimental to CFS integrity because mitotic entry with under-replicated sites triggers DNA breaks [39].

In agreement with the fact that Chk1 is not activated under moderate replication stress, the increase in chromosome instability observed in Chk1-depleted cells does not rely on its classical checkpoint function. Indeed, the frequencies of cells displaying ssDNA foci and breaks at CFSs are similar in checkpoint-proficient and in Chk1-depleted cells for similar fork speed reduction. In contrast, the frequencies of cells displaying ssDNA foci and chromosome breaks, notably at CFSs, increase considerably in cells depleted of ATR and submitted to moderate stress. This increase is accounted for neither by fork slowing nor by unscheduled mitotic entry. Noticeably, the specific increase in CFS breakage starts at the exact aphidicolin concentration that triggers chromatin recruitment of ATR in checkpoint-proficient cells. Together, these results confirm that ATR, and possibly other sensors and mediators of the pathway, plays a major role to prevent CFS instability under moderate fork slowing.

These results do not fit with a previous model postulating that, upon replication stress, helicases tend to travel uncoupled from polymerases along CFS, giving rise to long stretches of ssDNA. In sub-regions able to adopt secondary structures, ssDNA would evolve into fork barriers that cause DNA breaks. In this model, checkpoint-proficient cells are supposed to be protected against these deleterious events because local accumulation of ssDNA triggers the ATR signalling pathway, resulting in delayed mitotic onset and activation of the repair machinery [1]. We show here that under moderate stress conditions, similar to those used to induce breaks at CFSs, forks stall and/or collapse in ATR-deficient cells at the same frequency along *FRA3B* as in the bulk genome. These results support data previously obtained in checkpoint-proficient cells showing that forks do not encounter sequence-specific obstacles along the sites [2]. In addition, we show here that, in ATR-deficient cells, ssDNA foci result from Mre11-dependent resection of collapsed forks, suggesting that long stretches of ssDNA are a consequence rather than a cause of CFS instability. Together, these results strongly argue against the model above. In contrast, the remarkable sensitivity of CFSs to moderate replication stress in ATR-deficient cells is well explained by paucity in initiation events along large regions nested in the sites [2], [3]. Indeed, the lack of backup initiation events might prevent fast rescue of forks collapsing at random and normal frequency along CFSs, favouring extensive resection. This conclusion is supported by the fact that co-localization of ssDNA and CFSs increases specifically in the cell type in which a given site displays paucity in initiation events, namely *FRA3B* in lymphocytes and 3q13.3 in fibroblasts [2], [3].

Tumor suppressor genes are rarely mutated in sporadic cancers, notably in preneoplasic lesions [5]. Surprisingly, chromosome instability has been repeatedly observed in cells of these lesions, while DDR activation should block their proliferation. We show here in two cellular models that a moderate reduction of fork speed does not fully activate the DDR, fails to block mitotic entry and elicits breaks at under-replicated CFSs. Strikingly, some of the genes hosted by CFSs behave as tumor suppressor genes in human and mouse models [40]. *FRA3B*, for example, hosts the FHIT gene that has been involved in the cellular response to DNA damage. Thus, FRA3B expression not only triggers local instability but also favors genome-wide DNA damages through FHIT inactivation [41], [42]. The origin of the replication stress leading to CFS expression in pre-neoplastic lesions is still debated. It has been proposed to result from oncogene activation [7], from alteration of metabolic process or from exposure to environmental stress [42], [43]. Whatever the cause of CFS instability, we propose that imperfect repair of these damages in cells of pre-neoplastic lesions creates a pool of cells committed to further instability, from which the selection of cells with mutations affecting genes causally implicated in cancer development is facilitated.

## Materials and Methods

### Cell culture and transfection

Lymphoblastoid cells were grown in RPMI 1640+GlutaMAX-I medium (GIBCO) and MRC-5 cells in MEM plus Earle's salts without L-glutamine medium (GIBCO), 1% MEM nonessential amino acids (GIBCO), 1 mM sodium pyruvate (GIBCO) and 2 mM L-glutamine (GIBCO). In addition, all cells were grown with 10% foetal calf serum (PAN-Biotech GmbH) and 100 µg/mL of penicillin and streptomycin (GIBCO). For transfections, 2×10^6^ lymphoblastoid cells were resuspended in 100 µL of Nucleofector C solution (Lonza Cologne AG) with 0.6 µM RNAi and transfected with the Z-001 program according to manufacturer's instructions. MRC-5 cells were resuspended in Nucleofector R solution (Lonza Cologne AG) and transfected with the V-020 program. A mixture of 3 RNAi directed against ATR (HSS100876, HSS100877, HSS100878, Invitrogen) or Chk1 (HSS101854, HSS101855, HSS101856, Invitrogen) was used for transfection. The AllStars Negative Control siRNA (1027281, QIAGEN) was used for NONsi.

### DNA combing and image acquisition

Combing was performed as described [2], [44], [45]. An epifluorescence microscope (Axio Imager.Z2; Carl Zeiss) equipped with a 63× objective lens (PL APO, NA 1.4 Oil DIC M27) connected to a charge-coupled device camera (Cool-SNAP HQ2; Roper Scientific), and MetaMorph software (Roper Scientific) was used for image acquisition.

### Immunochemistry and metaphase spreading

Metaphase spreading and DNA fluorescent in situ hybridization for *FRA3B* detection were performed as described [39]. BACs were selected from the human genome project RP11 library. *FRA3B* has been assigned to band 3p14.2 and was probed with BAC 641C17. Cells were spread on slide with a cytospin (Shandon) and processed for immunostaining as previously described [46]. Combined immunofluorescence and DNA fluorescent in situ hybridization was performed according to Chaumeil *et al.* [47]. Microscopic images were acquired using an upright motorized microscope (Axio Imager.Z2; Carl Zeiss). Acquisitions were performed using an oil immersion objective 100× (PL APO, NA 1.46 Oil DIC M27) and a high-sensitive cooled interlined CCD camera (Cool-SNAP HQ2; Roper Scientific). For colocalization studies, rapid and precise Z-positioning was accomplished by a piezoelectric motor (P-725.1CD ; Physik Instrumente) mounted underneath the objective lens. Image stacks were acquired without camera binning, with a plane spacing of 0.2 µm, and MetaMorph software (Roper Scientific) was used for image acquisition.

### Cell extracts

Chromatin fractionation was performed as described [48]. For total extract of cells in S-phase, DNA was stained with 5 µg/ml Hoechst 33342 (Molecular Probes). Cells were sorted with a standard FACSVantage DiVa (Becton Dickinson Immunocytometry Systems, San Jose, CA) equipped with a 488 nm laser used at 250 mW and a multiline UV (351–363 nm) laser used at 200 mW. Linear Hoechst fluorescence was acquired using a 424/44 filter. Doublets were excluded using pulse area vs width. 10^5^ sorted cells were washed in cold PBS, re-suspended in 50 µL 1X SDS sample buffer (New England BioLabs Inc.), sonicated 5 min (Bioruptor - Diagenode) and boiled before loading for western blotting.

### Mitotic flow

Cells were treated with aphidicolin and nocodazole as indicated and washed with buffer GM (1.1 g/L Glucose, 8 g/L NaCl, 0.4 g/L KCl, 0.37 g/L Na_2_HPO_4_, 0.15 g/L KHPO_4_, 0.5 mM EDTA). The cells were re-suspended in 1 mL of buffer GM and fixed by addition of 3 mL of ethanol 100%. The cell pellet was incubated with a mouse anti-MPM2 (Mitotic Protein Monoclonal 2) antibody (05-368, Upstate) in buffer PBT (PBS 1X, 1% BSA and 0.05% Tween20), washed with PBS and incubated with a goat Alexa488-conjugated anti-mouse antibody (Molecular Probes) in PBT. Cells were washed with PBS and finally re-suspended in PBS containing 50 ng/µL propidium iodide and 40 µg/mL RNase A (USB). The quality of the mitotic cell preparations was verified by FACScan analysis of MPM2-positive cells with CellQuest software (Beckton Dickinson).

### Antibodies

The following primary antibodies were used in this study: mouse anti-PCNA (PC10) antibody (MAB424, Chemicon International), goat anti-ATR (N-19) antibody (sc-1887, Santa Cruz Biotechnology, Inc.), rabbit anti-Claspin antibody (A300-266A, Bethyl Laboratories, Inc.), mouse anti-Rad17 (sc-17761, Santa Cruz Biotechnology, Inc.), rabbit anti-phospho-Rad17 (Ser645) antibody (#3421, Cell Signaling Technology), rabbit anti-Rad9 (sc-8324, Santa Cruz Biotechnology, Inc.), goat anti-Hus1 antibody (sc-30543, Santa Cruz Biotechnology, Inc.), rabbit anti-RPA32 antibody (GTX70258, GeneTex, Inc.), mouse anti-Chk1 antibody (sc-8408, Santa Cruz Biotechnology, Inc.), rabbit anti-phospho-Chk1 (Ser317) antibody (#2344, Cell Signaling Technology), rabbit anti-phospho-Chk1 (Ser345) antibody (#2348, Cell Signaling Technology), rabbit γ-H2AX (phospho S139) antibody (ab2893, Abcam). In immunochemistry experiments, primary antibodies were visualized with Alexa594-conjugated goat anti-rabbit and Alexa488-conjugated goat anti-mouse antibodies (Molecular Probes). Detection of ssDNA was performed as previously described [49], with a rat anti-BrdU (OBT0030, AbD Serotec) and Alexa594-conjugated goat anti-rat antibodies (Molecular Probes).

## Supporting Information

Figure S1 Distributions of fork speed (kb per min) in JEFF cells. Cells were treated as indicated. Horizontal orange lines represent the medians of fork distributions. Median values are indicated above the distributions.
(PDF)

Figure S2 Study of DDR activation in MRC-5 cells. (A) Fork speed (kb/min) in cells treated for 4 h with the indicated aphidicolin concentrations. The mean replication speed in each condition is presented. Asterisks indicate that fork speed cannot be measured. (B) Western blot analysis of chromatin extracts showing chromatin recruitment of ATR after 4 h of treatment with the indicated aphidicolin concentrations. PCNA: loading control. (C) Western blot detection of Chk1-Ser317 and Ser345 phosphorylations in total extracts of cells treated for 4 h with the indicated concentrations of aphidicolin or 1 h with HU 1 mM.
(PDF)

Figure S3 Study of DDR activation in JEFF cells. (A) Left panel, western blot detection of Chk1-Ser317 phosphorylation in total extracts of FACS-sorted S-phase cells, treated as indicated. Right panel, quantification of Chk1-Ser317P from 3 independent experiments. Results (mean ± s.e.m) are expressed as percentage of the level of phosphorylation found in cells treated with HU 1 mM. (B) Immunofluorescence detection of chromatin bound PCNA (green) and Chk1 phosphorylated on Ser317 (red) in untreated cells (-) and in cells treated as indicated. Nuclei are counterstained with DAPI. (C) Western blot analysis of chromatin bound ATM and ATM-Ser1981 phosphorylation from exponentially growing cells, untreated (-) or treated as indicated. (D) Detection of γ-H2AX in total extracts of FACS-sorted S-phase cells treated as indicated. (E) Percentage of cells in mitosis 48 h post-transfection with a NONsi RNA or siRNAs specific to ATR or Chk1, in cell populations treated for 4 h with the indicated aphidicolin concentrations followed by 4 h with nocodazole and aphidicolin in the presence of 100 µM z-VAD-fmk. (F) Correlations between replication fork speed and the percentage of cells in mitosis. The data presented in A and Figure 5C were used to plot the percentage of cells in metaphase against fork speed.
(PDF)

Figure S4 Examples of JEFF cell analysis by cytogenetics approaches and 3D analysis of the relative localization of FRA3B and ssDNA foci in ATR-depleted JEFF cells treated with aphidicolin 0.3 µM. (A) Metaphase plates obtained in the indicated condition of transfection and treatment, and stained with DAPI. Red arrows point to broken chromosomes. (B) FISH with BAC 641C17 probing for *FRA3B*. Cells were treated with 0.3 µM aphidicolin. Left: DAPI alone. Right: DAPI (blue) with FISH signal (red). The arrow points to a chromosome broken at *FRA3B*. (C) FISH signal with BAC 641C17 (blue) and ssDNA (red). The yellow arrows point to FISH signals co-localizing with ssDNA foci.
(PDF)

Figure S5 Schematic representation of all fibres analyzed in ATR-depleted JEFF cells treated with aphidicolin 0.3 µM. Upper panel: *FHIT* gene (orange box) with its exons (E1 to E10); the Morse code used for FISH comprises 31 probes (green bars) organized in six motifs (a, b, c, i, d and e) that identify a 1.6 Mb-long region. Lower panels: DNA fibres bearing Morse code motifs and replication signals (newly synthesized DNA labelled *in vivo* with IdU then CldU, respectively revealed in blue and red). A schematic representation of the DNA fibres (grey) and of replication tracks (IdU in blue and CldU in red) is shown below each fibre. Asterisks indicate asymmetrical forks.
(PDF)
