# Supplementary figures and images for: Stepwise Activation of the ATR Signaling Pathway upon Increasing Replication Stress Impacts Fragile Site Integrity
Source: PLoS Genet. 2013 Jul 18;9(7):e1003643. doi: 10.1371/journal.pgen.1003643 (PMC3715430; doi:10.1371/journal.pgen.1003643)

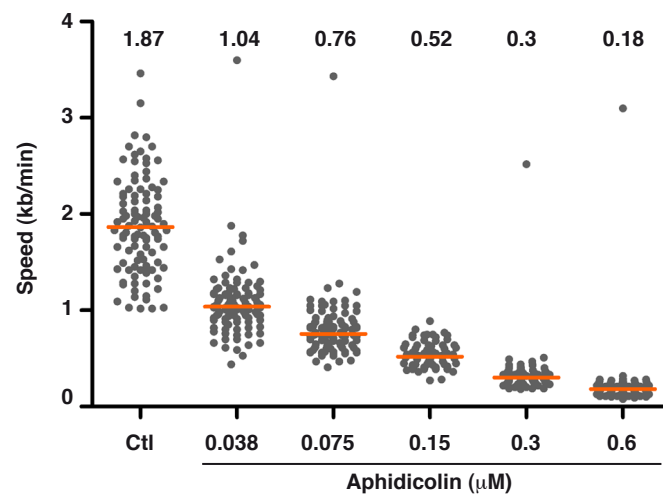

Supplement: Figure S1 — Distributions of fork speed (kb per min) in JEFF cells. Cells were treated as indicated. Horizontal orange lines represent the medians of fork distributions. Median values are indicated above the distributions. (PDF) [file pgen.1003643.s001.pdf]

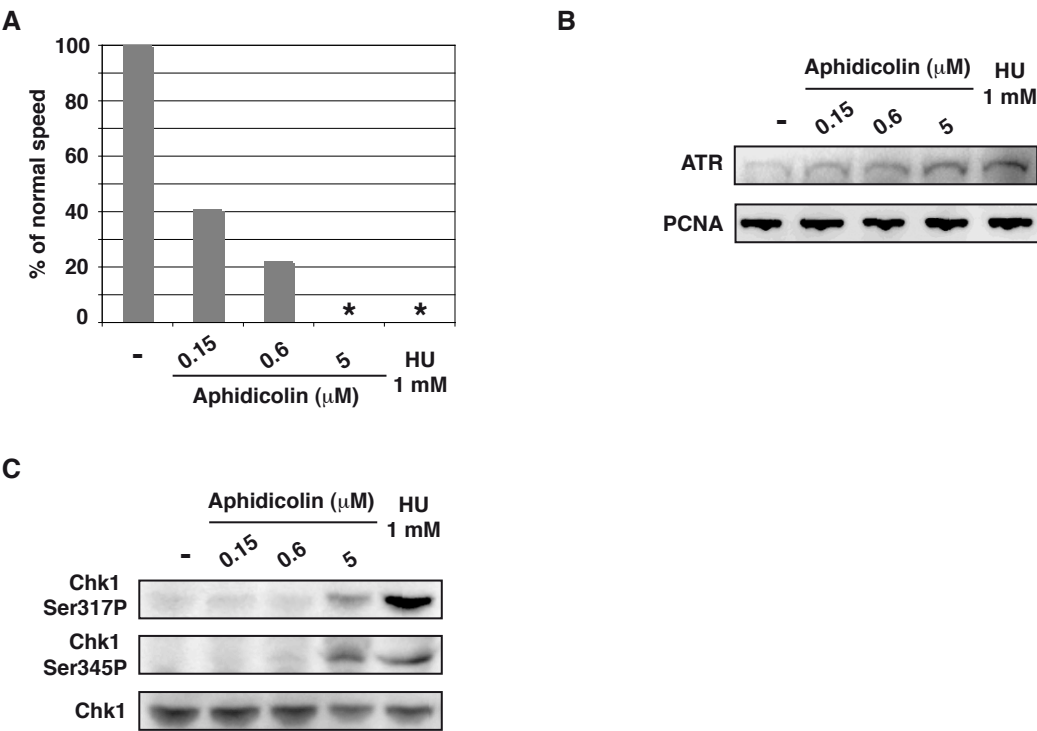

Supplement: Figure S2 — Study of DDR activation in MRC-5 cells. (A) Fork speed (kb/min) in cells treated for 4 h with the indicated aphidicolin concentrations. The mean replication speed in each condition is presented. Asterisks indicate that fork speed cannot be measured. (B) Western blot analysis of chromatin extracts showing chromatin recruitment of ATR after 4 h of treatment with the indicated aphidicolin concentrations. PCNA: loading control. (C) Western blot detection of Chk1-Ser317 and Ser345 phosphorylations in total extracts of cells treated for 4 h with the indicated concentrations of aphidicolin or 1 h with HU 1 mM. (PDF) [file pgen.1003643.s002.pdf]

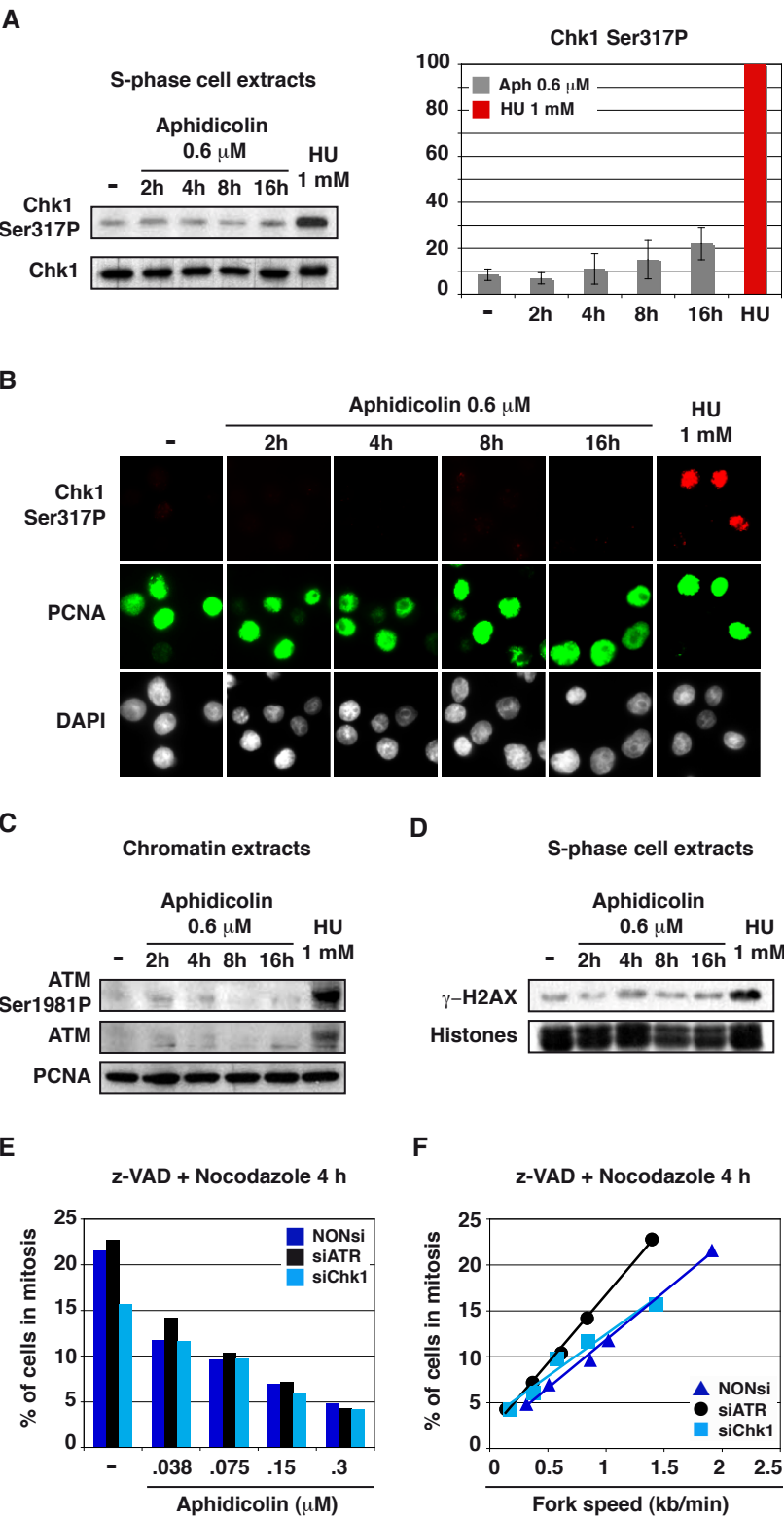

Supplement: Figure S3 — Study of DDR activation in JEFF cells. (A) Left panel, western blot detection of Chk1-Ser317 phosphorylation in total extracts of FACS-sorted S-phase cells, treated as indicated. Right panel, quantification of Chk1-Ser317P from 3 independent experiments. Results (mean ± s.e.m) are expressed as percentage of the level of phosphorylation found in cells treated with HU 1 mM. (B) Immunofluorescence detection of chromatin bound PCNA (green) and Chk1 phosphorylated on Ser317 (red) in untreated cells (-) and in cells treated as indicated. Nuclei are counterstained with DAPI. (C) Western blot analysis of chromatin bound ATM and ATM-Ser1981 phosphorylation from exponentially growing cells, untreated (-) or treated as indicated. (D) Detection of γ-H2AX in total extracts of FACS-sorted S-phase cells treated as indicated. (E) Percentage of cells in mitosis 48 h post-transfection with a NONsi RNA or siRNAs specific to ATR or Chk1, in cell populations treated for 4 h with the indicated aphidicolin concentrations followed by 4 h with nocodazole and aphidicolin in the presence of 100 µM z-VAD-fmk. (F) Correlations between replication fork speed and the percentage of cells in mitosis. The data presented in A and Figure 5C were used to plot the percentage of cells in metaphase against fork speed. (PDF) [file pgen.1003643.s003.pdf]

A

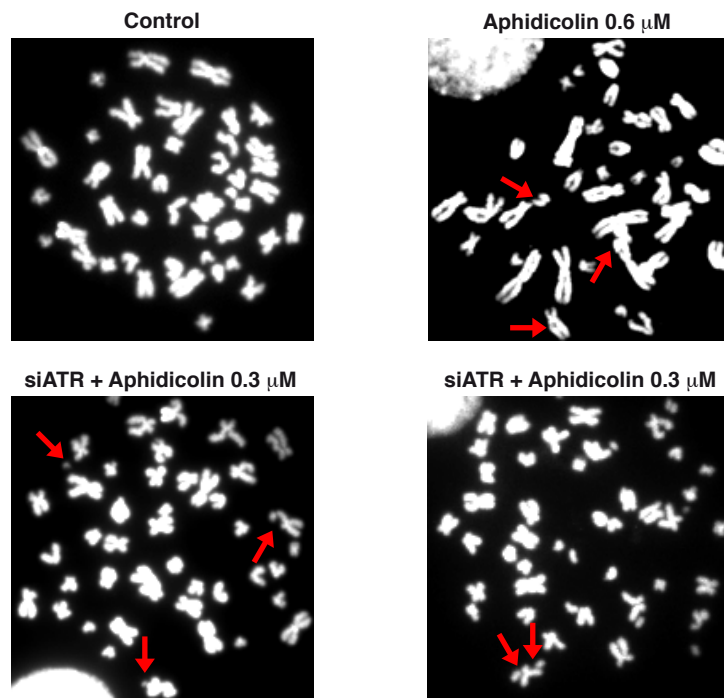

B

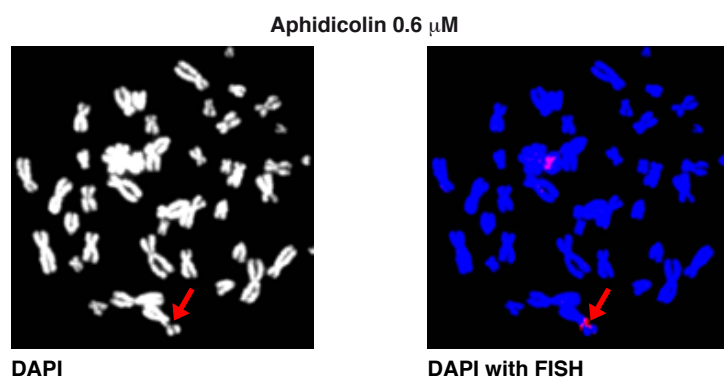

C

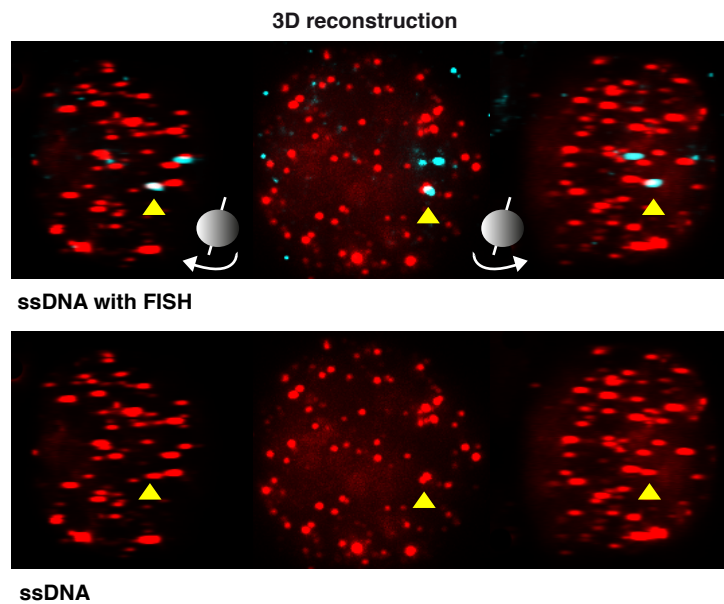

Supplement: Figure S4 — Examples of JEFF cell analysis by cytogenetics approaches and 3D analysis of the relative localization of FRA3B and ssDNA foci in ATR-depleted JEFF cells treated with aphidicolin 0.3 µM. (A) Metaphase plates obtained in the indicated condition of transfection and treatment, and stained with DAPI. Red arrows point to broken chromosomes. (B) FISH with BAC 641C17 probing for FRA3B. Cells were treated with 0.3 µM aphidicolin. Left: DAPI alone. Right: DAPI (blue) with FISH signal (red). The arrow points to a chromosome broken at FRA3B. (C) FISH signal with BAC 641C17 (blue) and ssDNA (red). The yellow arrows point to FISH signals co-localizing with ssDNA foci. (PDF) [file pgen.1003643.s004.pdf]

ATR-depleted JEFF cells treated with aphidicolin 0.3  $\mu$ M

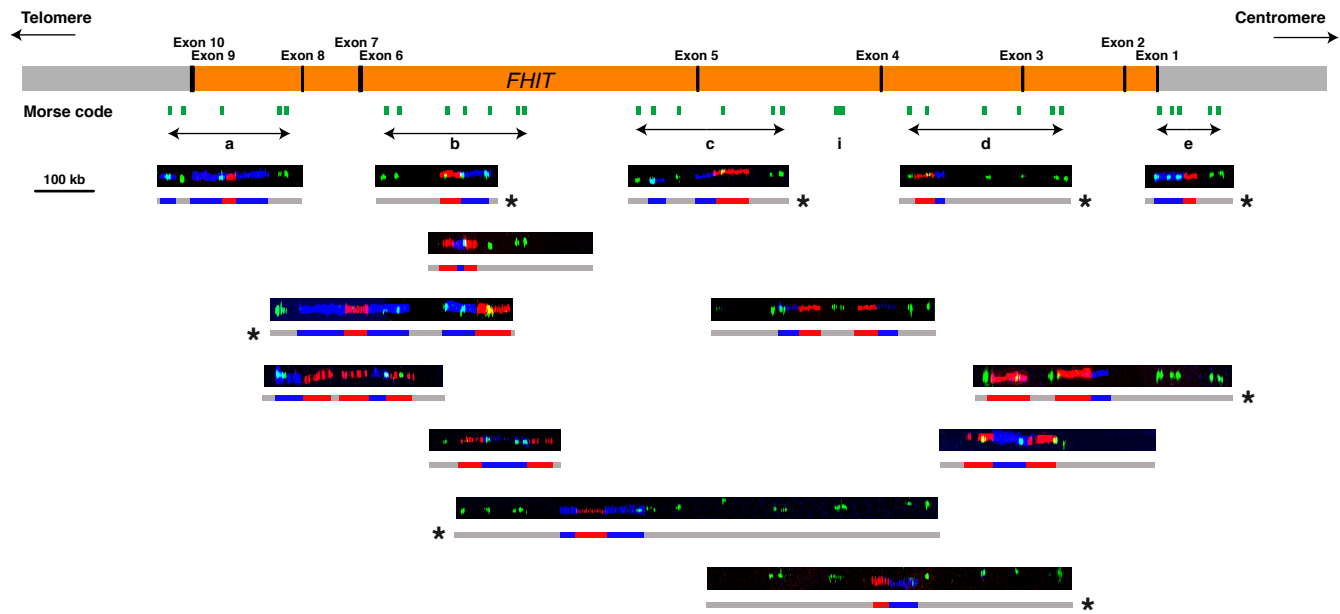

Supplement: Figure S5 — Schematic representation of all fibres analyzed in ATR-depleted JEFF cells treated with aphidicolin 0.3 µM. Upper panel: FHIT gene (orange box) with its exons (E1 to E10); the Morse code used for FISH comprises 31 probes (green bars) organized in six motifs (a, b, c, i, d and e) that identify a 1.6 Mb-long region. Lower panels: DNA fibres bearing Morse code motifs and replication signals (newly synthesized DNA labelled in vivo with IdU then CldU, respectively revealed in blue and red). A schematic representation of the DNA fibres (grey) and of replication tracks (IdU in blue and CldU in red) is shown below each fibre. Asterisks indicate asymmetrical forks. (PDF) [file pgen.1003643.s005.pdf]
